# Increased Level of Extracellular ATP at Tumor Sites: *In Vivo* Imaging with Plasma Membrane Luciferase

**DOI:** 10.1371/journal.pone.0002599

**Published:** 2008-07-09

**Authors:** Patrizia Pellegatti, Lizzia Raffaghello, Giovanna Bianchi, Federica Piccardi, Vito Pistoia, Francesco Di Virgilio

**Affiliations:** 1 Department of Experimental and Diagnostic Medicine, Section of General Pathology, Interdisciplinary Center for the Study of Inflammation (ICSI), University of Ferrara, Ferrara, Italy; 2 Laboratory of Oncology, Giannina Gaslini Institute, Genoa, Italy; 3 Animal Research Facility, Istituto Tumori, Genoa, Italy; Massachusetts General Hospital and Harvard Medical School, United States of America

## Abstract

**Background:**

There is growing awareness that tumour cells build up a “self-advantageous” microenvironment that reduces effectiveness of anti-tumour immune response. While many different immunosuppressive mechanisms are likely to come into play, recent evidence suggests that extracellular adenosine acting at A2A receptors may have a major role in down-modulating the immune response as cancerous tissues contain elevated levels of adenosine and adenosine break-down products. While there is no doubt that all cells possess plasma membrane adenosine transporters that mediate adenosine uptake and may also allow its release, it is now clear that most of extracellularly-generated adenosine originates from the catabolism of extracellular ATP.

**Methodology/Principal Findings:**

Measurement of extracellular ATP is generally performed in cell supernatants by HPLC or soluble luciferin-luciferase assay, thus it generally turns out to be laborious and inaccurate. We have engineered a chimeric plasma membrane-targeted luciferase that allows *in vivo* real-time imaging of extracellular ATP. With this novel probe we have measured the ATP concentration within the tumour microenvironment of several experimentally-induced tumours.

**Conclusions/Significance:**

Our results show that ATP in the tumour interstitium is in the hundrends micromolar range, while it is basically undetectable in healthy tissues. Here we show that a chimeric plasma membrane-targeted luciferase allows *in vivo* detection of high extracellular ATP concentration at tumour sites. On the contrary, tumour-free tissues show undetectable extracellular ATP levels. Extracellular ATP may be crucial for the tumour not only as a stimulus for growth but also as a source of an immunosuppressive agent such as adenosine. Our approach offers a new tool for the investigation of the biochemical composition of tumour milieu and for development of novel therapies based on the modulation of extracellular purine-based signalling.

## Introduction

While it is well established that ATP is an ubiquitous extracellular messenger, simple and reliable techniques to measure its concentration in the interstitium are missing. A few years ago Dubyak and co-workers [1] showed that a protein-A-luciferase chimera is a suitable probe of pericellular ATP in cells sensitized with IgG, and more recently Dale and Gourine have introduced a biosensor technique [2], [3]. These measurements clearly demonstrated that the concentration of ATP in the pericellular space is at least ten-fold higher than in the bulk solution and may reach tens of micromoles/liter. However, it would be of great biological interest to exploit the strong luciferase-dependent luminescence to image extracellular ATP *in vivo*. This might be especially valuable for a deeper understanding of tumour-host interactions, as it is increasingly appreciated that tumours build up a specific microenvironment that affects crucially host immune response and cancer cell proliferation and dissemination [4], [5].

Recent data show that adenosine concentration is much higher in the interstitium of solid tumours compared to healthy tissues [6], an observation with far-reaching implications given the immunosuppressive activity of this ATP metabolite [6], [7]. Although most cells express adenosine transporters, there are few doubts that a main source of extracellular adenosine is hydrolysis of extracellular ATP by cell surface ecto-ATPases [8], [9]. Thus, it would be of interest to be able to monitor in real time extracellular ATP levels in healthy and diseased tissues. To this aim, we have engineered a chimeric luciferase-folate receptor construct in which the luciferase cDNA was appended to targeting sequences (leader sequence and GPI anchor) derived from the folate receptor [10]. The chimeric protein is targeted to, and retained at the plasma membrane thus detecting ATP in the aqueous layer close to the cell surface.

We have now used this novel probe, named pmeLUC (plasma membrane luciferase), to generate stably transfected HEK293 cells (HEK293-pmeLUC cells) that act as *in vivo* sensors of the ATP concentration in the tumour microenvironment.

## Results

**Figure 1:**
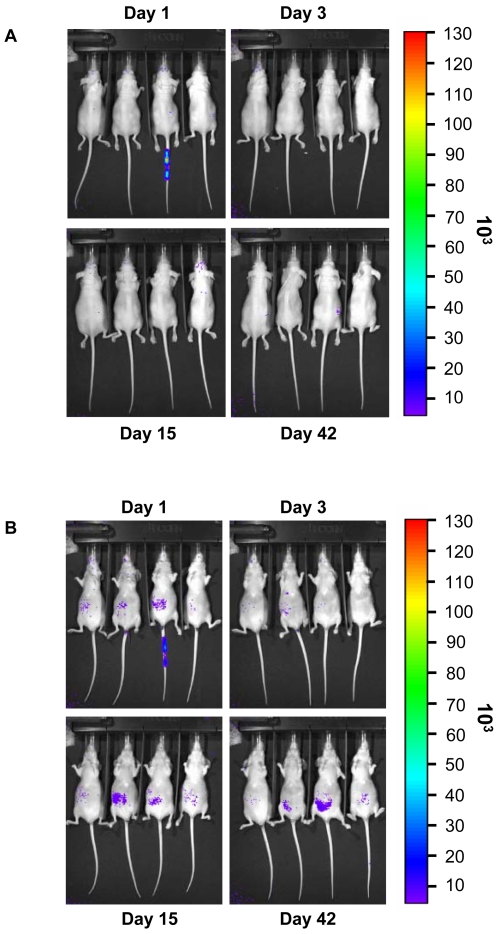
Imaging of healthy nude mice i.v. injected with HEK293-pmeLUC cells. Mice were injected i.v. with 2×10^6^ HEK293-pmeLUC cells. D-luciferin was administred intraperitoneally. Images were captured 15 minutes after D-luciferin administration. Acquisition time was 3 min, and images were acquired over 2 months every 2 days both from the dorsal (A) and the ventral (B) view. The earliest time point was at 15 min after D-luciferin injection. Only images taken after 1, 3, 15 and 42 days are shown.

**Figure 2:**
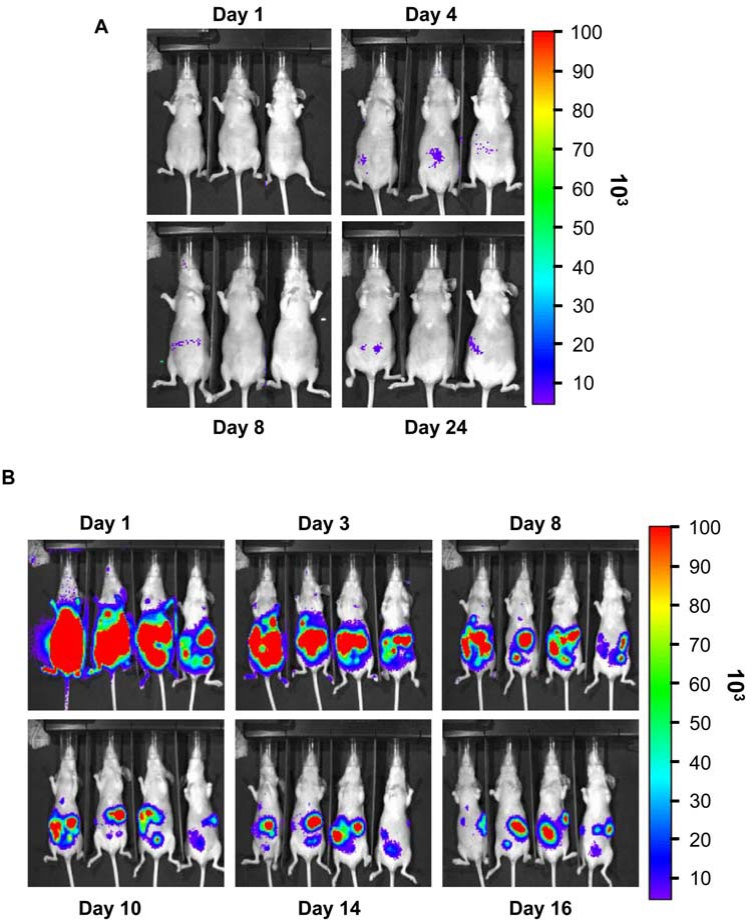
Imaging of tumour-bearing nude mice injected with HEK293-pmeLUC cells. (A) Healthy nude mice were injected i.p. with 2×10^6^ HEK293-pmeLUC cells and monitored for 3 months (only 4 time points are shown). (B) Nude mice were injected i.p. with the human ovarian carcinoma cell line OVCAR-3 (1.5×10^6^). Twenty days post-implantation, HEK293-pmeLUC cells (2×10^6^) were i.p. injected, followed by D-luciferin. OVCAR-3-bearing mice were monitored over 16 days, and were then euthanized according to bioethical regulations.

We anticipated that HEK293-pmeLUC cells might function as *in vivo* reporters of the interstitial ATP concentration in healthy and diseased tissues. As shown in figure 1, i.v. inoculation of HEK293-pmeLUC cells in healthy nude mice caused a modest, barely detectable, increase in luminescence, except for the third mouse from the left, which showed a strong signal at site of injection in the tail, likely due to tissue damage and ATP release during the injection. The tail luminescence signal gradually disappeared, and for up to 42 days no additional luminescence foci appeared throughout the body in any of the mice, whether imaged in a dorsal (figure 1A) or abdominal (figure 1B) projection. In three experiments involving 10 mice, we observed a faint, transient abdominal luminescence only in two animals. HEK293-pmeLUC cells were then i.p. inoculated (figure 2A). In this case also no significant light emission was detected for up to 24 days of observation. These experiments show that HEK293-pmeLUC cells do not detect significant extracellular ATP levels when inoculated i.v. or i.p. into healthy animals, unless tissue damage occurs, as shown by the single mouse in figure 1A.

**Figure 3:**
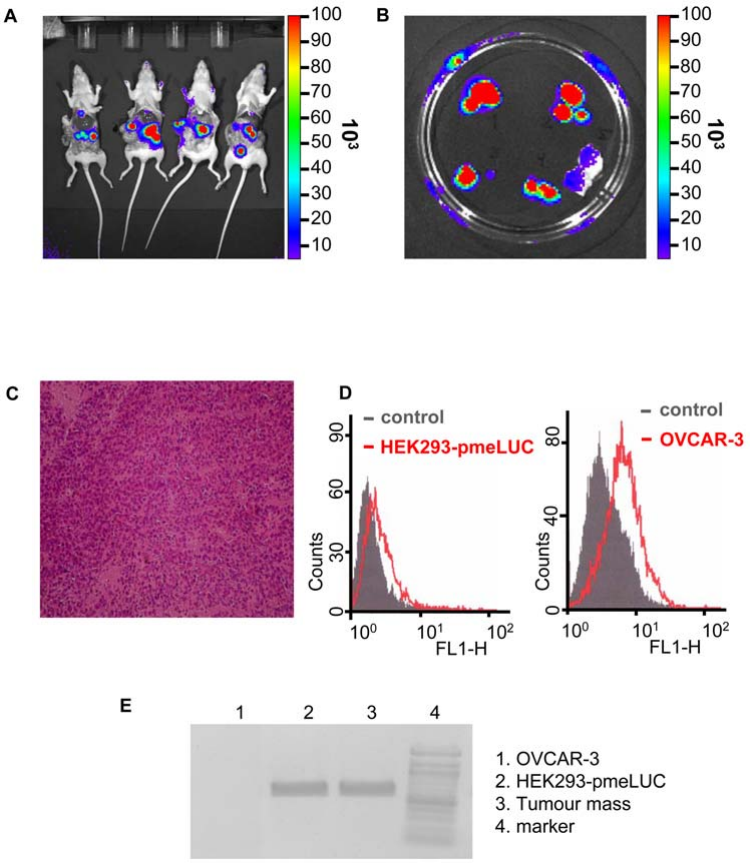
HEK293-pmeLUC cells localize to tumour foci. (A) Mice were euthanized, the peritoneal cavity was opened and imaged. (B) Single tumour foci were excised and luminescence recorded. (C) Tissue section from one of the tumour foci stained with haematoxylin/eosin. (D) Cytofluorimetric analysis of cells isolated from tumour foci. Staining was performed with monoclonal FITC-labeled rabbit anti-luciferase (red trace on the left panel) or anti-CD51 (red trace on the right panel) Abs. Irrelevant IgG_1_ Ab (grey trace on both the right and left panel) was used as control. (E) Expression of pmeLUC mRNA in tumour foci excised from mice injected with OVCAR-3 and HEK293-pmeLUC cells. OVCAR-3 and HEK293-pmeLUC cells grown in culture are shown as controls. RT-PCR was performed as described in Materials and Methods.

However, it may well be that this failure is due to unsuitability of HEK293-pmeLUC cells to report extracellular ATP in vivo. To clarify this issue, we inoculated HEK293-pmeLUC cells into mice bearing the OVCAR-3 human ovarian carcinoma (figure 2B). In tumour-bearing mice luminescence was very bright shortly after i.p. inoculation (first image was acquired 15 sec after injection, not shown), likely signalling the ATP-rich inflammatory environment within the peritoneal cavity, then attenuated slightly and localized to discrete abdominal foci (figure 2B). At day 16 after injection, mice were sacrificed and subjected to post-mortem analysis. Luminometric examination of the peritoneal cavity revealed dissemination of numerous light-emitting metastasis throughout the peritoneum (figure 3A). To identify the cell types present in the metastasis, some of the peritoneal masses were excised (figure 3B), and subjected to histological analysis (figure 3C), which revealed the typical morphology of an ovarian carcinoma. To further characterize the cell types present, the tumour masses were homogenized, stained with Abs raised against either luciferase (to detect HEK293-pmeLUC cells) or the OVCAR-3-specific antigen CD51 (to detect ovarian carcinoma cells), and analyzed by cytofluorimetry (figure 3D). As a final proof of the presence of infiltrating HEK293-pmeLUC cells within the tumour masses, tumour homogenates were assayed for the presence of pmeLUC message (figure 3E). The pmeLUC message was very abundant in the tumour masses (figure 3E, lane 3). As controls we run samples from cultured HEK293-pmeLUC cells, which showed a signal of intensity comparable to that of tumours homogenates, and from cultured OVCAR-3 cells, which were not surprisingly negative for pmeLUC expression. This is unequivocal proof that HEK293-pmeLUC cells infiltrate the OVCAR-3 tumour masses.

**Figure 4:**
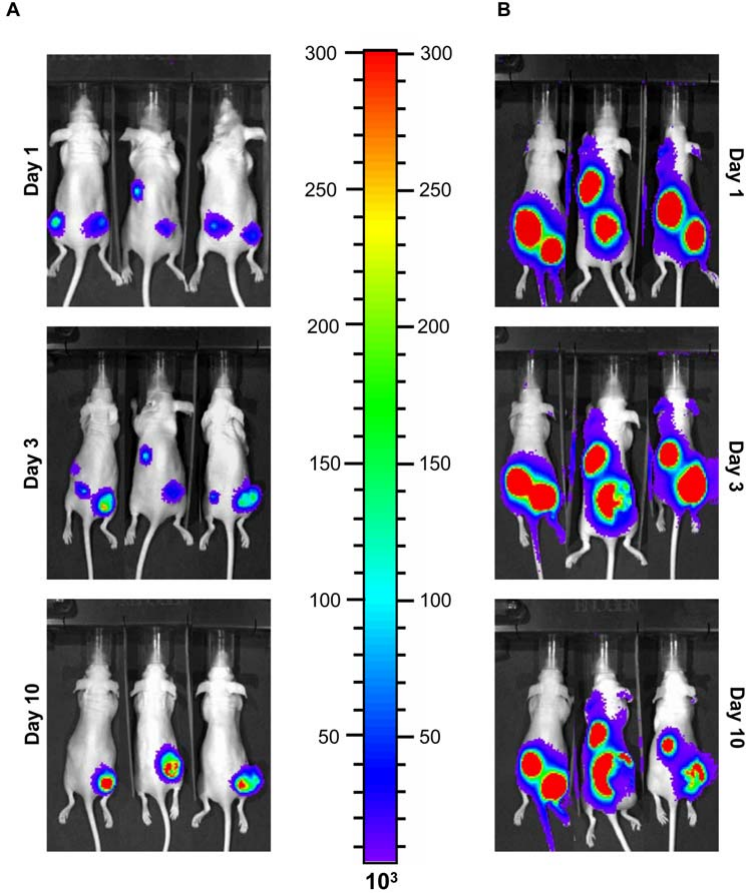
Comparison of luminescence emission from HEK293-pmeLUC and HEK293-cytLUC cells injected into MZ2-MEL melanoma and healthy tissue. Nude mice were inoculated with MZ2-MEL cells in the right dorsal hip. After about 20 days, when the tumour was about 1–1.5×1–1.5 cm size, HEK293-pmeLUC (A) or HEK293-cytLUC (B) cells were injected s.c. into the tumour bearing (right) or healthy (left) site.

These experiments provide three valuable pieces of information: a) the ATP concentration in the extracellular environment is very low in healthy tissues, certainly below the threshold for HEK293-pmeLUC detection (about 1–5 µM); b) HEK293-pmeLUC cells infiltrate ovarian carcinoma growing in the peritoneal cavity and report ATP in the tumour microenvironment; c) light emission from tumours is very bright, close to saturation of the luciferase signal, thus the intratumour extracellular ATP concentration must be orders of magnitude higher than that in the healthy tissue. However, there are some important caveats. Exogenous cancer cells are known to chemotact towards, infiltrate and even proliferate within tumour masses [11], thus it is possible that the strong luminescence signal detected in the ovarian carcinoma-bearing mice is not due to the increased ATP concentration in the tumour microenvironment, but rather to the increased local density of HEK293-pmeLUC cells. If this were the case, the higher local luciferase concentration would certainly cause enhanced light emission. To address this issue, we inoculated the same amount of HEK293-pmeLUC cells (2×10^6^) s.c. into an established MZ2-MEL human melanoma, or controlateral healthy tissue. Under these conditions, since the local tissue concentration of luciferase is the same at both sites, changes in light emission should reflect only differences in ATP levels. As shown in figure 4A, light emission, which was very strong from both sites 1 day after injection, increased steadily from the tumour-bearing side, while progressively faded from the healthy side (compare day 3 to 1), to disappear completely at day 10. It is very likely that the initial luminescence signal originating from both sites was due to the injection trauma, as clearly shown in figure 1. As tissue injury healed, luminescence decreased in the healthy but not tumour-bearing site, indicating a stable, higher interstitial ATP level, in agreement with the experiments shown in figure 2.

**Figure 5:**
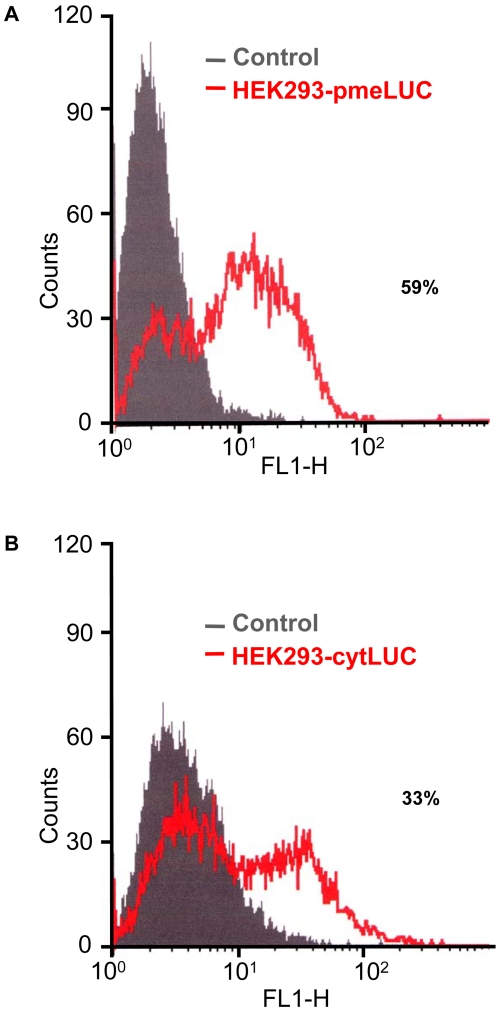
Luciferase expression by HEK293-pmeLUC and HEK293-cytLUC. Plasma membrane (A) or cytosolic (B) luciferase expression was analyzed with a specific anti-luciferase Ab (see Figure 3). An isotype-matched control mAb was used as control. Luciferase expression is reported as percentage of positive cells.

**Figure 6:**
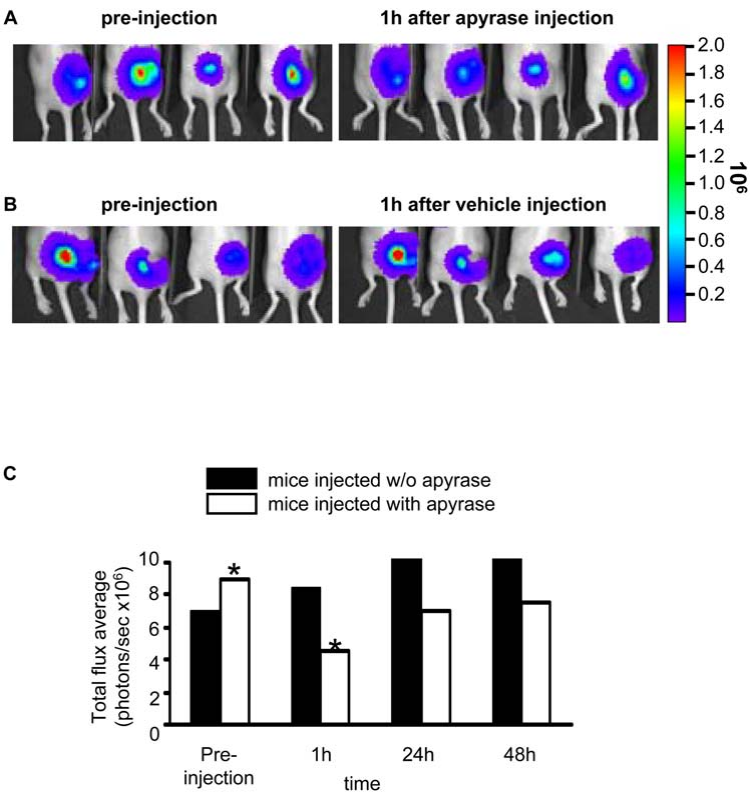
Sensitivity of HEK293-pmeLUC cells to apyrase. A melanoma was induced as described in Figure 4, and inoculated with HEK293-pmeLUC cells 20 days after implant. Apyrase (20 U in 40 µl of serum free DMEM ) or vehicle (serum free DMEM) was injected into the tumour four days later. Bioluminescence was recorded as indicated. (A) Apyrase-injected, or (B) vehicle-injected mice. (C) Quantitative analysis of light emission from region-of-interest. Data are averages of measurements from 4 mice (n = 4). * p<0.05 (paired *t*-test).

**Figure 7:**
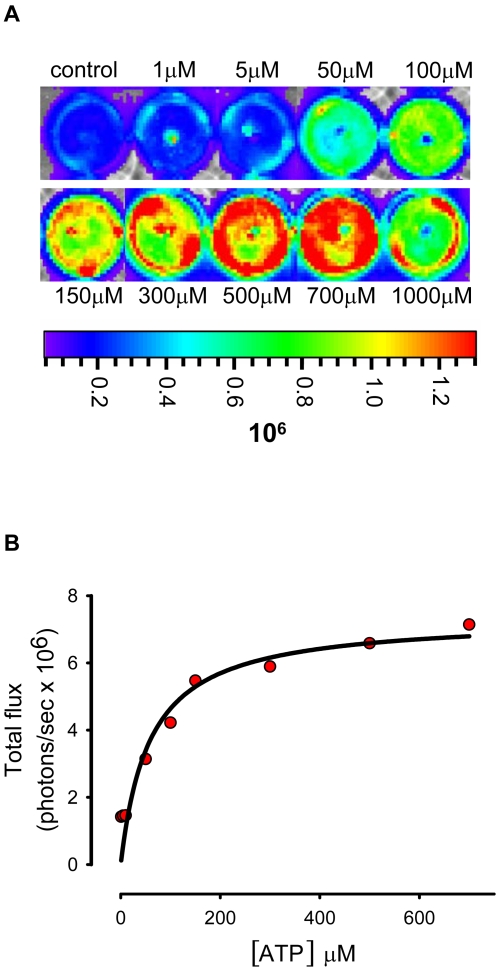
In vitro calibration of HEK293-pmeLUC bioluminescence. (A) HEK293-pmeLUC cells, 7×10^4^ per well, were incubated in DMEM medium and challenged with increasing concentrations of extracellular ATP and luminescence acquired with the IVIS luminometer for 1 min. In (B) luminescence (total flux expressed as photon per sec) was expressed as a function of the ATP concentration. Control well contained only DMEM medium. D-luciferin was added to all wells at a concentration of 60 µg/ml.

However, it might be objected that although the same amount of cells are inoculated into the tumour and the healthy tissue, HEK293-pmeLUC are retained longer at the site of intratumour injection, while on the contrary they might migrate away from the site of injection when inoculated into healthy tissue. This would lead to a dilution of the luciferase signal from the healthy tissue. To rule out this possibility, we engineered HEK293 cells with a luciferase construct encoding a luciferase lacking the plasma membrane-targeting sequence, and therefore localized in the cytosol (HEK293-cytLUC). This luciferase monitors intracellular but is insensitive to extracellular ATP. FACS analysis showed that HEK293-cytLUC and HEK293-pmeLUC express luciferase to the same level (figure 5, A and B), thus they allow a careful comparison of luminescence emission. As shown in figure 4B, HEK293-cytLUC emitted a strong, long lasting, light signal from both healthy and tumour-bearing sites. In contrast to mice injected with HEK293-pmeLUC, the signal from healthy tissue injected with HEK293-cytLUC did not disappear and was still very strong at day 10, indicating that inoculated cells were retained close to the site of injection. To further validate HEK293-pmeLUC cells as ATP reporters, we tested their sensitivity to the ATP-hydrolyzing enzyme apyrase. Apyrase was injected into the tumour site 4 days after HEK293-pmeLUC cells, and luminescence emission monitored during 48 h (figure 6). We anticipated that if HEK293-pmeLUC cells were faithful indicators the extracellular ATP levels, apyrase injection should cause a drop in luminescence. This was indeed the case, as shown by imaging (figure 6, A and B) and quantitative photon counting of the region-of-interest (figure 6C). Luminescence emission was decreased by apyrase by about 50%, and recovered steadily to pre-apyrase levels within 2 days, likely as a consequence of enzyme diffusion from or inactivation at the injection site. Vehicle injection on the contrary caused as expected a damage-induced luminescence increase. Finally, we performed an *in vitro* calibration of HEK293-pmeLUC cell luminescence with the IVIS luminometer which indicated an ATP concentration in the tumour interstitium in the hundred micromolar range, or possibly higher, as the signal saturated above 700 µM ATP (figure 7).

## Discussion

There is growing awareness that the tumour microenvironment has a key role in supporting tumour growth and in dictating the rules of host-tumour interaction [12], [5]. The tumour may wield host response by inducing the formation of protected niches that allow survival of cancer stem cells and their differentiation into mature cancer cells [13]. The tumour microenvironment which includes infiltrating inflammatory cells as well as stromal cells, is responsible for creating conditions that hinder the effectiveness of the host immune response and lead to immunoevasion, or even to tumour progression [14]. The biochemical composition of the tumour microenvironment is poorly known, but it is understood that it may profoundly change depending on tumor type and the host immunocompetence [15].

Depending on the tight balance between tumour-induced or tumour-released immunosuppressive factors and host-derived immunoactivating factors the microenvironment creates favourable or unfavourable conditions for tumour growth. This generates a network of facilitating or inhibitory interactions the effect of which is extremely difficult to anticipate. In this context hypoxic conditions that characterize several tumours may be an important component of the mechanism of tumour protection. Hypoxia causes the activation of hypoxia-inducible factor 1 α (HIF-1α) and accumulation of extracellular adenosine. Both factors are in principle very important in supporting tumour growth as HIF-1α controls angiogenesis and adenosine exerts a profound immunosuppressive activity, thus protecting the tumour from inflammatory cells. Recent data show that solid tumours have a gradient of adenosine concentration from the centre to the periphery, higher than the surrounding healthy tissue [6]. In addition, many tumours over-express enzymes involved in the catabolism of extracellular nucleotides and in the generation of adenosine [16]. Furthemore, glioblastoma cells injected in vivo together with apyrase show a reduced ability to produce tumours [17]. Accumulation of adenosine into the tumour microenvironment does not only protect tumour cells from the immune response, but may also exert a trophic effect on the tumour itself by stimulating endothelial cell proliferation and angiogenesis [18], [19], [20].

Although cells express carriers that may mediate adenosine translocation into the extracellular milieu, most extracellular adenosine is generated at the expenses of extracellular ATP via extracellular nucleotidases (ecto-ATPases and 5′-nucleotidase) [21], [22]. However, ATP in the tumour microenvironment is important not only as a source of adenosine but also for its intrinsic activity. In fact ATP itself modulates inflammation by triggering IL-1 maturation and release, dendritic cell differentiation by inducing a Th2-skewing phenotype and cell proliferation or cell death, depending on the concentrations and the activation of individual P2 receptors [23]. In addition, its has been recently shown that ATP causes shedding of metalloproteases (MMP9) [24] and expression of indoleamine oxygenase [25]; both activities may be very relevant for tumour progression as MMP9 release facilitates tumour invasion while indoleamine oxygenase has immunosuppressive activity.

Bioluminescence imaging is increasingly recognized as a powerful tool to study in vivo transcriptional regulation, signal transduction, activation of cancer-specific genes. So far, luciferase has been almost exclusively used as an intracellular reporter, to monitor the activity of specific transcriptional activators such as for example the estrogen receptor [26] or NF-κB [27]. In this study we show that cells engineered with luciferase can be also used to probe the extracellular space and to analyze the biochemical composition of the tumour microenvironment. Furthermore, since an increased ATP concentration is a feature of inflammation, engineering inflammatory cells with pmeLUC will make possible *in vivo* imaging of inflammation. Finally, since appending proper target sequences to luciferase may allow targeting to specific regions of the plasma membrane, we anticipate that pmeLUC may even allow to probe changes in the extracellular ATP concentration at restricted sites of cell-to-cell interaction.

## Materials and Methods

### Cell lines

Human embryonic kidney cells (HEK293) were purchased from the American Type Culture Collection (Rockville, MA, USA). Cells were cultured in Dulbecco's modified eagle medium (DMEM) (Sigma Aldrich, Milano, Italy) supplemented with 10% fetal bovine serum (FBS), penicillin G (50 units/ml), and streptomycin sulfate (50 µg/ml). The OVCAR-3 human ovarian carcinoma and MZ2-MEL human melanoma cell lines (purchased from Dr. T. Boon, Ludwig Institute for Cancer Research, Brussels, Belgium), were grown as a monolayer at 37°C in a humidified 5% CO_2_ atmosphere in RPMI 1640 (Life Technologies, Grand Island, NY) supplemented with 10% FBS, penicillin G (50 units/ml), and streptomycin sulfate (50 µg/ml) (Sigma).

### Luciferase constructs

Targeting of luciferase to the extracellular aspect of the plasma membrane (pmeLUC) was achieved by adding to luciferase cDNA a N-terminal endoplasmic reticulum import sequence (leader sequence), and a C-terminal glycophosphatidylinositol anchor sequence (GPI sequence) of the human folate receptor. These minimal targeting and anchor signals allow localization of luciferase on the outer side of the plasma membrane. The luciferase used was *Photinus pyralis* luciferase modified for optimizing transcriptional activity in eukaryotic cells (Promega Italia SrL, Milano, Italy). Luciferase cDNA was amplified from a modified pGL3-control plasmid (kind gift of Dr Guy Rutter, University of Bristol, UK) using the following primers 5′ - CCC TGC AGA TGG AAG ACG CCA AAA ACA TAA AGA AAG G - 3′ (corresponding to the sequence encoding amino acids 1–9 of luciferase; *PstI* site underlined) and 5′ - GCT GCA GCC ACG GCG ATC TTT CCG CCC TTC TTG G - 3′ (including amino acids 542–549 of luciferase cDNA without the stop codon; *PstI* site underlined). The PCR product was transferred to pBSK+ vector (Agilent-Stratagene, Santa Clara CA, USA), digested with the enzyme *PstI* and inserted in right frame between a *PstI* fragment encoding the complete N–terminal leader sequence of the human folate receptor (26 aa) fused with myc tag (10 aa) and a *PstI* fragment of the GPI anchor protein (28 aa), to generate the pmeLUC construct. The whole final construct was excised by a *NotI/XhoI* or *XbaI* digestion, and cloned into the expression vectors pcDNA3 or VR1012, as described previously (10).

Untargeted cytosolic luciferase (cytLUC) was based on the pGLP3-basic vector (Promega) modified by insertion of cytomegalovirus promoter excised from a pcDNA3 vector (Invitrogen SrL Milano, Italy).

### Transfection of HEK293 cells and stable clones selection

HEK293 transfection with plasma membrane-targeted luciferase (pmeLUC) or cytosolic luciferase (cytLUC) was carried out with Lipofectamine 2000 (Invitrogen, San Giuliano Milanese, Italy). Selection was performed in the presence of G418 as previously described [10].

### Real time *in vivo* imaging

*In vivo* bioluminescent imaging was performed with a ultra low-noise, high sensitivity cooled CCD camera, mounted on a light-tight imaging chamber (IVIS 100 System™, Xenogen, Roissy, France). Tracking, monitoring and quantification of signals were controlled by the acquisition and analysis software Living Image® (Xenogen). After cell inoculation, 150 mg/kg D-luciferin was i.p administered (3 mg/mouse), and the luminescence was captured from both dorsal and ventral views. D-luciferin was administered to anesthetized (1–3% isoflurane) animals 15 minutes before image acquisition. Anesthetized mice were then placed in the IVIS™ Imaging System and imaged. Three-four mice were imaged at each time. Regions of interest from displayed images were identified around the tumor sites and were quantified as total photon counts or photons/s using the Living Image® software. For i.v. inoculation, cells (2×10^6^/mouse) from a stable HEK293-pmeLUC clone were slowly injected into the tail vein of 5–6 weeks old athimic nude (*nude/nude*) mice. Animals were monitored every 3–4 days. For i.p. inoculation, nude mice were injected with 1.5×10^6^ OVCAR-3 cells suspended in 200 µl of FBS-free RPMI medium followed by i.p. inoculation of HEK293-pmeLUC cells (2×10^6^/mouse). Tumour-free nude mice were also inoculated with HEK293-pmeLUC cells as a control. Luminescence was monitored every 2 days for up to 16 (OVCAR-3 bearing mice injected with HEK293-pmeLUC) or 90 days (control mice). Tumour-bearing mice were sacrificed after 16 days according to bioethical regulations. For s.c. inoculation, a melanoma xenograft was induced on the right dorsal hip of nude mice by a injection of 8×10^6^ MZ2-MEL cells. Fifteen–twenty days after tumor inoculation, when the melanoma had reached a size of 1,5×1,5 cm, mice were subcutaneously injected with HEK293-pmeLUC cells in the left tumour-free, dorsal hip (control), and in the melanoma-bearing right dorsal hip. Mice were imaged every 2 days. Strict animal care procedures were in accordance with institutional guidelines in compliance with national and international laws and policies (European Economic Community Council Directive 86/109, OJL 358, Dec. 1, 1987 and NIH Guide for the Care and Use of Laboratory Animals). Mice used in these studies were 5- to 6-week-old *nude/nude* mice, weighing 18 to 25 g, purchased from Harlan Laboratories (Udine, Italy) and housed in sterile enclosures under specific virus and antigen-free conditions.

### *In vitro* and *ex vivo* imaging

For *in vitro* imaging, 7×10^4^ HEK293-pmeLUC cells were seeded into 12-well plates (Becton Dickinson Biosciences, Franklin Lakes, NJ, USA). 60 µg/well of D-luciferin (Xenogen) was added to each well 30 min before imaging. Imaging time was 3 min/plate. For *ex vivo* imaging, mice were handled as described above and no further luciferin was injected into the mice before necropsy. Tissues of interest were excised, placed into 10 cm tissue culture plate and acquired for 3 minutes. Tissues were then fixed in 10% formalin (Sigma) and subjected to histology.

### Histology

To confirm the presence of HEK293-pmeLUC, tumour mass were excised from the mice at necropsy and imaged right away. After bioluminescent analysis, tissue samples were fixed in 10% formalin solution (Sigma Aldrich) and embedded in paraffin. For histology, 5 µm thick sections were cut from formalin fixed, paraffin embedded, blocks and stained with hematoxylin/eosin.

### Analysis of pmeLUC/mRNA Expression

Total RNA was extracted with RNAspin Mini Isolation Kit (GE Healthcare, Milano, Italy) from OVCAR-3 cells (as a negative control), HEK293-pmeLUC stable clone (as positive control), and OVCAR-3 xenografts from mice inoculated with HEK293-pmeLUC. Primers for pmeLUC were the following: forward 5′-ATATGTGGATTTCGAGTCGTC-3′ and reverse 5′-GATGGATTCCAATTCAGCGGG-3′ (PCR product, 597 bp). To obtain cDNA, 200 ng of total RNA were transcribed with Access RT-PCR System (Promega, Milano, Italy). RT-PCR was performed using 10 µl of buffer AMV/Tfl Reaction buffer, 30 µl H_2_O DNAse/RNAse free, 2 µl of each primer (final concentration 20 picoM each), and 2 µl of MgSO_4_ (stock, 25 mM). Retrotranscription conditions were: 2 min at 95°C, then 1 cycle of 48°C for 55 min and 2 min at 95°C. Thirty five cycles of amplification followed: 94°C for 1 min, 56°C for 1 min, 72°C for 2 min), and a final elongation at 72°C for 8 min. PCR products were resolved by electrophoresis on a 1% agarose gel.

### Flow cytometry

For staining of intracellular luciferase (cytLUC), cells were fixed with 2% paraformaldehyde (Sigma Aldrich) at room temperature for 20 min, rinsed twice with permeabilization buffer (PBS, 1% FBS, 0.1% saponin; Sigma Aldrich), and incubated with a luciferase specific mAb (8–10 µg/5×10^5^ cells) (Abcam, Cambridge, UK) or an irrelevant mouse IgG_1_ (Serotec, Oxford, UK). Cells were then washed twice with permeabilization buffer, incubated with FITC-conjugated F(ab')_2_ fragments of rabbit anti-mouse IgG antibodies, washed twice in permeabilization buffer and resuspended in staining buffer before being analyzed by flow cytometry with a FACScan instrument (Becton Dickinson Biosciences). For surface staining, cells isolated from OVCAR-3 i. p. xenografts of mice inoculated with HEK293-pmeLUC were incubated with an optimal amount of luciferase specific mAb or with anti-CD51 (1 µg/5×10^5^ cells) (Becton Dickinson Biosciences). The primary antibody incubation was followed by an optimal amount of secondary antibody incubation (FITC-conjugated F(ab')_2_ fragments of rabbit anti-mouse Ig antibodies). Stained cells were analyzed by flow cytometry. Isotype- and subclass-matched mouse Ig were used as negative controls in all the experiments at a concentration of 100 ng/mL. Cell Quest software (Becton Dickinson Biosciences) was used for data analysis.

### Statistical analys

Statistical analysis was performed using the Stat View software package (SAS Institute Inc, Cary, NC, US). The data were analysed by the Student *t* test for paired samples. Statistical significance was assumed for p<0.05 (two tailed).
